# Drop‐offs in the isoniazid preventive therapy cascade among children living with HIV in western Kenya, 2015–2019

**DOI:** 10.1002/jia2.25939

**Published:** 2022-08-04

**Authors:** Dickens Otieno Onyango, Marianne A. B. van der Sande, Courtney M. Yuen, Jerphason Mecha, Daniel Matemo, Elizabeth Oele, John Kinuthia, Grace John‐Stewart, Sylvia M. LaCourse

**Affiliations:** ^1^ Kisumu County Department of Health Kisumu Kenya; ^2^ Institute of Tropical Medicine Antwerp Belgium; ^3^ Julius Global Health, Julius Center for Health Sciences and Primary Care University Medical Center Utrecht Utrecht the Netherlands; ^4^ Harvard Medical School Boston Massachusetts USA; ^5^ Department of Research and Programs Kenyatta National Hospital Nairobi Kenya; ^6^ Department of Global Health University of Washington Seattle Washington USA; ^7^ Division of Allergy and Infectious Diseases Department of Medicine, University of Washington Seattle Washington USA; ^8^ Department of Epidemiology University of Washington Seattle Washington USA; ^9^ Department of Pediatrics University of Washington Seattle Washington USA

**Keywords:** human immunodeficiency virus, Mycobacterium tuberculosis infection, preventive therapy, children, cascade, isoniazid

## Abstract

**Introduction:**

Isoniazid preventive therapy (IPT) can reduce the risk of tuberculosis (TB) in children living with HIV (CLHIV), but data on the outcomes of the IPT cascade in CLHIV are limited.

**Methods:**

We evaluated the IPT cascade among CLHIV aged <15 years and newly enrolled in HIV care in eight HIV clinics in western Kenya. Medical record data were abstracted from September 2015 through July 2019. We assessed the proportion of CLHIV completing TB symptom screening, IPT eligibility assessment, IPT initiation and completion. TB incidence rate was calculated stratified by IPT initiation and completion status. Risk factors for IPT non‐initiation and non‐completion were assessed using Poisson regression with generalized linear models.

**Results:**

Overall, 856 CLHIV were newly enrolled in HIV care, of whom 98% ([95% CI 97–99]; *n* = 841) underwent screening for TB symptoms and IPT eligibility. Of these, 13 (2%; 95% CI 1–3) were ineligible due to active TB and 828 (98%; 95% CI 97–99) were eligible. Five hundred and fifty‐nine (68%; 95% CI 64–71) of eligible CLHIV initiated IPT; median time to IPT initiation was 3.6 months (interquartile range [IQR] 0.5–10.2). Overall, 434 (78%; 95% CI 74–81) IPT initiators completed. Attending high‐volume HIV clinics (aRR = 2.82; 95% CI 1.20–6.62) was independently associated with IPT non‐initiation. IPT non‐initiation had a trend of being higher among those enrolled in the period 2017–2019 versus 2015–2016 (aRR = 1.91; 0.98–3.73) and those who were HIV virally non‐suppressed (aRR = 1.90; 95% CI 0.98–3.71). Being enrolled in 2017–2019 versus 2015–2016 (aRR = 1.40; 1.01–1.96) was independently associated with IPT non‐completion. By 24 months after IPT screening, TB incidence was four‐fold higher among eligible CLHIV who never initiated (8.1 per 1000 person years [PY]) compared to CLHIV who completed IPT (2.1 per 1000 PY; rate ratio [RR] = 3.85; 95% CI 1.08–17.15), with a similar trend among CLHIV who initiated but did not complete IPT (8.2/1000 PY; RR = 4.39; 95% CI 0.82–23.56).

**Conclusions:**

Despite high screening for eligibility, timely IPT initiation and completion were suboptimal among eligible CLHIV in this programmatic cohort. Targeted programmatic interventions are needed to address these drop‐offs from the IPT cascade by ensuring timely IPT initiation after ruling out active TB and enhancing completion of the 6‐month course to reduce TB in CLHIV.

## 1 INTRODUCTION

Tuberculosis (TB) is a leading cause of death among people living with HIV [1, 2] and a significant cause of morbidity and mortality in children living with HIV (CLHIV) [3, 4]. TB prevalence in Kenya was estimated to be 558 per 100,000 population in 2016 [5]. Childhood TB accounts for 9% of all TB notifications in Kenya, and nearly a third of children on TB treatment are co‐infected with HIV [6].

A 6‐month course of isoniazid preventive therapy (IPT) has been shown to reduce the risk of developing TB by up to 60% in children [7]. The implementation of IPT in CLHIV involves multiple steps, constituting a care cascade. These steps entail identifying eligible children, evaluation to rule out active TB, initiating them on IPT and completion of 6 months of IPT [8]. Barriers at any step of the IPT cascade lead to drop‐offs, thus reducing the potential benefit of IPT programs to reduce TB [8].

Although the Kenya Ministry of Health introduced IPT among CLHIV in 2014, rates of initiation remained low until September 2015 when a massive rollout began [9]. Data are limited on the programmatic performance of the IPT cascade among CLHIV, including longer‐term TB incidence in high TB burden countries. Most studies from high burden settings evaluated the IPT cascade among under‐five children who are contacts of people with TB and reported IPT initiation rates ranging from 2.3% to 100% and completion rates ranging from 0% to 95% [10]. Previous studies that evaluated the IPT cascade in Kenya focused primarily on adults with HIV [9, 11, 12].

We conducted this analysis among CLHIV who were newly enrolled in high‐ and moderate‐volume HIV clinics in Kisumu County, western Kenya to (1) determine the proportion of CLHIV completing key steps of the IPT cascade; (2) identify risk factors for IPT non‐initiation and non‐completion; and (3) compare the risk of TB among CLHIV who completed IPT versus those who did not.

## 2 METHODS

### 2.1 Study setting

The annual TB case notification rate in Kisumu County was 209 per 100,000 population in 2019; 52% of the notified cases were co‐infected with HIV [13]. HIV prevalence among adults in Kisumu County was estimated to be 17.5% in 2018, over thrice the national prevalence of 4.9%, while the prevalence among children aged <15 years was 0.7% [14]. HIV services in the County are offered within HIV clinics that are mainly based in high‐volume hospitals and follow national guidelines regarding TB screening and prevention [15]. CLHIV are enrolled in HIV clinics where they are initiated on antiretroviral therapy (ART) and followed up monthly. Screening for TB symptoms is conducted at baseline and during monthly HIV clinic follow‐up visits using an intensive case finding (ICF) questionnaire. CLHIV with cough of any duration, fever, night sweats or noticeable weight loss are evaluated for active TB. All CLHIV aged 12 months and over without TB symptoms and those with TB symptoms ones in whom active TB is ruled out upon further evaluation are eligible for IPT [16]. CLHIV who are initiated on IPT are followed up at regular intervals as determined by respective clinics, which are usually synchronized with ART follow‐up visits until they finish the 6‐month course. IPT follow‐up information among children who initiated IPT, including IPT completion outcomes at 6 months (completed IPT, lost to follow up, discontinued IPT, dead or transferred out) and TB status at 12, 18 and 24 months, is recorded in a paper‐based IPT register. For purposes of this analysis, transfer out was considered “non‐completion” due to a lack of further information. Other clinical information is recorded in electronic medical records that capture HIV treatment information, including viral load, opportunistic infections and the child's current care status (whether active, transferred out or lost to follow‐up). The medical charts are usually completed by nurses or attending clinicians. For this study, data were collected from eight HIV clinics in Kisumu County (Jaramogi Oginga Odinga Teaching and Referral Hospital, Kisumu County Hospital, Lumumba Sub‐county Hospital, Migosi Sub‐county Hospital, Rabuor Sub‐county Hospital, St. Monica Clinic, Ahero County Hospital and Chulaimbo County Hospital).

### 2.2 Study design

This was a retrospective cohort study based on a review of routinely collected data abstracted from the paper‐based registers and electronic medical records of CLHIV aged 12 months to <15 years who were newly enrolled in HIV care at the participating health facilities (including those who were transferred in from other facilities) from September 2015 through July 2019. Data abstraction occurred from November 2019 through November 2020. The data abstraction tool captured the following information: baseline demographic and clinical characteristics (date of HIV diagnosis, date of enrolment to HIV care, date of ART initiation, history of opportunistic infections, viral load at IPT initiation [within 6 months] or the most recent for non‐initiators [within 6 months of the most recent IPT eligibility evaluation], screening for TB symptoms, IPT initiation, monthly IPT follow‐up for 6 months, IPT outcome at 6 months and TB status at 12, 18 and 24 months after IPT initiation). IPT initiation and completion statuses were assessed based on documentation by attending clinicians in the medical records and IPT cards.

### 2.3 Data analysis

We assessed the cascade estimating proportions completing eligibility screening out of identified CLHIV, IPT initiation out of those who were eligible and IPT completion out of those who initiated IPT with 95% confidence intervals. For this analysis, CLHIV with a negative TB symptom screen, or if symptomatic, had active TB ruled out after further evaluation were considered to be eligible for IPT. Bivariate and multivariable analysis was conducted to determine the risk factors for IPT non‐initiation among eligible children and non‐completion among those who initiated using a modified Poisson regression. We considered both patient‐level and facility‐level risk factors. Factors with a *p*‐value of <0.10 on bivariate analysis were included in multivariate analysis. The most recent viral load results before IPT initiation (within 6 months) were included in the analysis. For IPT non‐initiators, viral load results obtained within 6 months of the most recent IPT eligibility evaluation were used. CLHIV with a viral load of less than 1000 copies per millilitre was considered virally suppressed. HIV clinics were categorized into moderate‐volume (<5000 active clients) and high‐volume (≥5000 active clients). Factors with a *p*‐value less than 0.05 were considered statistically significant in the multivariable analysis.

We compared TB incidence rates among three groups of CLHIV: (1) those who were eligible but never initiated IPT (IPT non‐initiators), (2) those who initiated but never completed IPT (IPT non‐completers) and (3) those who completed the 6‐month IPT course (IPT completers) as the reference group. TB incidence rates were calculated as the sum of new cases divided by the person‐years at risk while in care. Person‐years at risk was calculated as the interval from the last date of IPT eligibility screening to the date of TB diagnosis or the date of the last clinic visit or the date they transferred care to another facility, and rate ratios were calculated with 95% confidence intervals.

Data were analysed using Stata version 16 (StataCorp, College Station, TX, USA).

### 2.4 Ethical considerations

Ethical clearance was obtained from the University of Washington Institutional Review Board, Kenyatta National Hospital/University of Nairobi Ethics Review Committee, and Jaramogi Oginga Odinga Teaching and Referral Hospital Ethics Review Committee. Informed consent was waived since this study only involved abstraction and analysis of data that was collected as part of routine clinical care.

## 3 RESULTS

### 3.1 Demographic and clinical characteristics of participants

Overall, 856 CLHIV between the ages of 12 months and 14 years were newly enrolled in HIV care from September 2015 through July 2019. At enrolment, the median age was 5 years (interquartile range [IQR] 1.9–9.2) and 54% (*n* = 465) were female (Table 1). Seventy‐eight percent (*n* = 667) of the CLHIV were WHO Stage 1 or 2, 14% (*n* = 119) were WHO Stage 3 or 4, while 8% (*n* = 70) were missing information on WHO Stage. Ninety‐nine percent (*n* = 844) of CLHIV had a date of ART initiation. Of these, 78% (*n* = 657) were initiated on ART at enrolment. Among those who initiated ART post‐enrolment, the median time to ART initiation was 0.5 months (IQR = 0.2–2.3). Viral load was available for 58% (*n* = 497) of CLHIV. The median viral load at IPT initiation was 60 copies per millilitre (IQR = 0–1154); 71% (*n* = 373) were virally suppressed. Among non‐initiators, the median viral load within 6 months of the most recent IPT eligibility evaluation was 229.5 copies per millilitre (IQR = 70–8989.5); 56% (*n* = 18) were virally suppressed.

**Table 1:** Baseline demographic and clinical characteristics

Characteristic	Number	Percent (%)
Age at enrolment		
Median age in years (interquartile range)	5.0	1.9–9.2
12 months to 4 years	425	50
5–9 years	239	28
10–14 years	187	22
Sex		
Male	391	46
Female	465	54
Facility volume		
High‐volume (≥5000 active clients)	570	67
Moderate‐volume (<5000 active clients)	286	33
Year of enrolment		
2015–2016	500	58
2017–2019	356	42
WHO Stage		
Stage 1/2	667	79
Stage 3/4	119	14
Unknown	61	7
Timing of ART initiation (*N* = 844)		
At enrolment	652	77
Post enrolment	192	33
Median months to ART post enrolment (interquartile range)	0.5	0.2–2.3

### 3.2 TB screening and IPT eligibility evaluation

**Figure 1:**
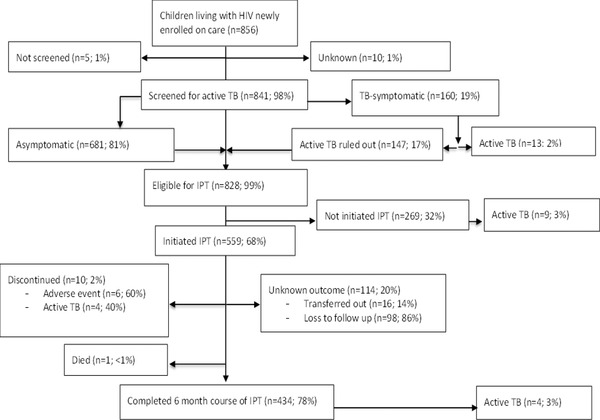
IPT cascade among CLHIV enrolled on HIV care, Kisumu County, western Kenya, 2015–2019.

Nearly, all (98% [95% CI 97–99]; *n* = 841) of the enrolled CLHIV were screened for IPT eligibility using the ICF tool (Figure 1); 2% (95% CI 1–3; *n* = 13) of those screened were diagnosed with active TB. Thus, 98% (95% CI 97–99; *n* = 828) of the CLHIV who underwent screening were eligible for IPT initiation.

### 3.3 IPT initiation

**Figure 2:**
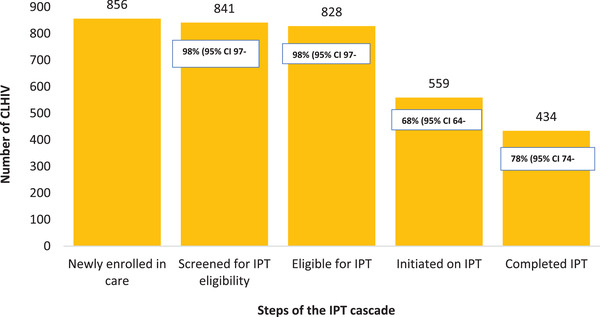
Children living with HIV completing steps of the isoniazid preventive therapy cascade, Kisumu County, western Kenya, 2015–2019. *Percentages reflect the proportion of participants from the previous step completing the step.

IPT was initiated in 68% (95% CI 64–71; *n* = 559) of eligible CLHIV (Figure 2). Of these children, 63% (95% CI 58–67; *n* = 350) started within 6 months of enrolment into HIV care, 15% (95% CI 12–18; *n* = 84) within 7–12 months and 19% (95% CI 16–23; *n* = 108) after 12 months. The date of IPT initiation was unknown for 3% (*n* = 17) of CLHIV who initiated IPT. Almost all (99%; *n* = 554) were on ART at IPT initiation; one participant was not on ART when IPT was initiated, while the ART status at IPT initiation of four participants was unknown.

### 3.4 IPT completion

Of 559 CLHIV who initiated IPT, 78% (95% CI 74–81; *n* = 434) completed the 6‐month course. IPT was discontinued in 2% (*n* = 10); six had adverse events, while four were diagnosed with active TB. One child died of an unknown cause within 2 months of IPT initiation. IPT outcomes were unknown in 20% (*n* = 114) of CLHIV who initiated; of these, 86% (*n* = 98) were lost to follow‐up, while 14% (*n* = 16) were transferred out to other health facilities. The median number of months on IPT was 1.5 (IQR 1.0–2.8) among those who discontinued, 3.2 (IQR 2.9–6.2) among those who were lost to follow‐up and 2.1 (IQR 1.0–4.9) among those who transferred out.

### 3.5 Risk factors for IPT non‐initiation and non‐completion

In bivariate analysis, younger age, later year of enrolment, viral load non‐suppression and higher HIV clinic volume were associated with non‐initiation of IPT among eligible CLHIV at a *p*‐value <0.10 level and thus included in the multivariable model (Table 2). In multivariable analysis adjusting for factors with a *p*‐value of <0.1 in bivariate analysis, IPT non‐initiation was nearly twice higher among those enrolled between 2017 and 2019 than those enrolled between 2015 and 2016 (aRR = 1.91; 95% CI 0.98–3.73), had a trend to be 90% higher among virally non‐suppressed CLHIV than virally suppressed (aRR = 1.90; 95% CI 0.98–3.71) and almost thrice as high among CLHIV attending high‐volume HIV clinics than among those attending lower volume clinics (aRR = 2.82; 95% CI 1.20–6.62).

**Table 2:** Factors associated with non‐initiation and non‐completion of IPT in newly enrolled CLHIV in western Kenya

	IPT initiation among eligible CLHIV *n* = 828					IPT completion among initiators *n* = 559				
Characteristic	Initiated *N* (Row %) (*N* = 559)	Not initiated *N* (Row %) (*N* = 269)	RR (95% CI)	Adjusted^b^ RR (95% CI)	*p*‐value	Completed *N* (Row %) (*N* = 434)	Never completed *N* (Row %) (*N* = 109)	RR (95% CI)	Adjusted^b^ RR (95% CI)	*p*‐value
Age at enrolment										
1–4 years	254 (62)	153 (38)	1.36 (1.12–1.66)	1.32 (0.68–2.57)	0.39	199 (81)	46 (19)	0.88 (0.63–1.24)	–	
5–14 years	302 (72)	115 (28)	Ref			232 (79)	63 (24)	Ref		
Sex										
Male	255 (68)	118 (32)	Ref			199 (81)	47 (19)	Ref		
Female	304 (67)	151 (33)	1.05 (0.86–1.28)	–		235 (79)	62 (21)	1.09 (0.78–1.53)	–	
HIV clinic volume^a^										
High‐volume	337 (61)	214 (39)	1.96 (1.51–2.53)	2.82 (1.20–6.62)	0.02	268 (82)	58 (18)	0.76 (0.54–1.06)	0.77 (0.55–1.08)	0.14
Moderate‐volume	222 (80)	55 (20)	Ref	Ref		166 (76)	51 (24)	Ref	Ref	
Year of enrolment										
2015–2016	350 (72)	134 (28)	Ref	Ref		285 (83)	60 (17)	Ref	Ref	
2017–2019	209 (61)	135 (39)	1.42 (1.17–1.72)	1.91 (0.98–3.73)	0.06	149 (75)	49 (25)	1.42 (1.02–1.99)	1.40 (1.01–1.95)	0.05
WHO Stage at enrolment										
Stage 1/2	473 (72)	180 (28)	1.02 (0.73–1.42)	–		367 (80)	93 (20)	1.31 (0.76–2.28)	–	
Stage 3/4	81 (73)	30 (27)	Ref			66 (85)	12 (15)	Ref		
Unknown	4 (7)	55 (93)	3.44 (2.52–4.72)	–		1 (25)	3 (75)	4.88 (2.26–10.52)	–	
Viral load at IPT initiation^c^										
Suppressed	330 (95)	18 (5)	Ref	Ref		290 (88)	38 (12)	Ref	Ref	
Non‐suppressed	132 (90)	14 (10)	1.85 (0.95–3.63)	1.90 (0.98–3.71)	0.06	108 (83)	22 (27)	1.46(0.90–2.37)	–	
Opportunistic infections										
Yes	27 (59)	19 (41)	1.29 (0.90–1.85)			20 (74)	7 (26)	1.32 (0.68–2.56)	–	–
No	530 (68)	249 (32)	Ref			413 (80)	101 (20)	Ref		
Had respiratory symptoms during screening										
Yes	106 (72)	41 (28)	0.84 (0.64–1.12)	–		79 (79)	21 (21)	1.06 (0.69–1.62)		–
No	448 (67)	221 (33)	Ref			351 (80)	87 (20)	Ref		
Time to IPT initiation										
<3 months						177 (77)	53 (23)	Ref		
3–6 months						69 (79)	18 (21)	1.11 (0.69–1.79)		
>6 months						173 (83)	36 (17)	0.83 (0.50–1.38)		

^a^ Low volume—<5000 active clients; high‐volume—≥ 5000 active clients.

^b^ Adjusted for factors that with a *p*‐value <0.1 during bivariate analysis.

^c^ Viral load suppression—less than 1000 copies per millilitre.

Among CLHIV who initiated IPT, year of enrolment and HIV clinic volume were associated with non‐completion of IPT in bivariate analysis at a *p*‐value <0.10 level and thus included in the multivariable model. In multivariable analysis, IPT non‐completion was 40% higher among those enrolled between 2017 and 2019 than those enrolled between 2015 and 2016 (aRR = 1.40; 95% CI 1.01–1.96).

### 3.6 TB incidence by 24 months after enrolment in HIV care

Overall, 30 CLHIV were diagnosed with active TB, including 13 with prevalent TB who were diagnosed at screening for IPT eligibility. Seventeen new cases were diagnosed after enrolment with an incidence of 4.9/1000 person‐years (Table 3); 53% (*n* = 9) occurred among CLHIV who were eligible but never initiated IPT, 24% (*n* = 4) among those who initiated IPT but did not complete the 6‐month course and 24% (*n* = 4) occurred among those who completed the 6‐month course. By 24 months after IPT screening, TB incidence was four‐fold higher among eligible CLHIV who never initiated (8.1 per 1000 person‐years [PY]) compared to CLHIV who completed IPT (2.1 per 1000 PY; rate ratio = 3.85; 95% CI 1.08–17.15), with a similar trend among CLHIV who initiated but did not complete IPT (8.2/1000 PY; rate ratio = 4.39 95% CI 0.82–23.56).

**Table 3:** Cumulative TB incidence per 1000 child years by 24 months after IPT eligibility evaluation

IPT initiation/completion status	TB diagnosis	Person‐time (years)	TB rate/1000 child years (95% CI)	Rate ratio (95% CI)
IPT eligible but never initiated	9	1108.8	8.1 (4.2–15.6)	3.85 (1.08–17.15)
Initiated but never completed IPT	4	433.3	9.2 (3.5–24.6)	4.39 (0.82–23.56)
Completed IPT	4	1901.5	2.1 (0.8–5.6)	Ref
Total	17	3443.6	4.9 (3.0–7.8)	

## 4 DISCUSSION

In this retrospective review of medical records of CLHIV in large HIV care programs following intensive scale‐up of IPT in Kenya, we found high levels of screening for IPT eligibility, but suboptimal levels of IPT initiation and completion. IPT non‐initiation among eligible CLHIV was associated with attending large referral hospitals for HIV care. There was a trend among those enrolled between 2017 and 2019 compared to earlier periods and those with HIV viral non‐suppression. IPT non‐completion was associated with being enrolled between 2017 and 2019 compared to earlier periods.

Although TB preventive therapy (TPT) is critical to reducing morbidity and mortality associated with TB in people living with HIV, it remains underutilized, especially in high TB/HIV burden settings, including sub‐Saharan Africa (SSA) [17]. Our results add to the scant evidence on the programmatic performance of the TPT cascade in CLHIV from SSA. Barriers that have been identified to contribute to losses in the TPT cascade [18] could be similar across several SSA countries. Thus, our programmatic evaluation data of the TPT cascade could inform programmatic interventions in other countries that seek to improve the uptake and completion of this life‐saving intervention in CLHIV.

Our findings, based on a large cohort of CLHIV aged 1–14 years from western Kenya (a region known for its high TB/HIV prevalence), contribute to the limited literature on IPT initiation and completion among CLHIV from high TB/HIV burden countries. The uptake of IPT among CLHIV in Kenya still falls short of the global target of >90% [19] and could be lagging behind that in adults living with HIV as reported by studies from other high TB prevalence countries [20, 21]. These findings strengthen the evidence from previous Kenyan studies that reported suboptimal IPT initiation among CLHIV, although those studies were conducted in regions of the country with lower TB/HIV prevalence, were based on small sample sizes and did not include all age groups [12, 22, 23].

Additionally, in this study, a substantial proportion of IPT initiators (40%) were initiated after being on HIV care for over 6 months. Suboptimal or delayed IPT initiation in this study could be due to health system barriers, which have been documented in previous studies, including difficulties in ruling out active TB especially in CLHIV with respiratory symptoms [24], the fear of adverse reactions to isoniazid (INH) [22], poor health provider adherence to IPT guidelines [25], frequent INH stockouts and concerns about inducing resistance to INH [26]. Our finding that CLHIV in high‐volume referral hospitals were almost three times less likely to initiate IPT is similar to a finding by Karanja et al. that people living with HIV (PLHIV) in level 4 and 5 hospitals were less likely to complete IPT compared to lower‐level facilities [11]. Such high‐volume hospitals have more complex service delivery models that challenge the integration of TB/HIV services [27]. The lower IPT initiation between the period 2017 and 2019 compared to 2015–2016 was probably due to a greater programmatic focus during the rapid scale‐up phase (2015–2016). Additionally, rapid scale‐up occasioned intermittent stockouts of 100 milligrams INH pills (paediatric formulation) from 2017 onwards thus slowing down uptake. In this study, there was a trend of virally non‐suppressed CLHIV being less likely to initiate IPT suggesting that they could have been sicker at enrolment, thus their providers may not have felt confident in ruling out active TB. The impact of caregiver barriers, such as the cost of travel for IPT services and the perception of minimal risk in asymptomatic children [28], may have also contributed.

There are limited data on outcomes of IPT among CLHIV in other high HIV/TB settings in SSA. In a prospective cohort study that enrolled 66 CLHIV from a paediatric clinic in Tanzania, 74% completed IPT within 10 months [29]. A study that evaluated programmatic data among people living with HIV attending two clinics in Kinshasa, the Democratic Republic of Congo included 546 CLHIV on IPT, 87% of whom were reported to have completed the 6‐month course [30]. IPT completion in this study (78%) was similar to a study conducted at Kenyatta National Hospital (82%) in Kenya [22]. Together, these findings suggest that a substantial proportion of CLHIV do not complete the 6‐month course of IPT and thus continue to be at risk of developing TB. However, IPT completion in our study could have been higher than reported given that 14% of CLHIV transferred out to other facilities where they may have completed. IPT completion in CLHIV may be improved when IPT refills are synchronized with ART follow‐up visits [31, 32]. In our study, HIV viral non‐suppression showed a trend for association with non‐initiation and non‐completion of IPT and is perhaps due to these CLHIV and their caretakers already having difficulty adhering to ART [33]. Virally non‐suppressed CLHIV may require additional support to initiate and complete IPT. IPT completion could also be improved by introducing shorter TPT regimens [34, 35]. The Kenya Ministry of Health has recommended shorter regimens for under‐five childhood contacts of bacteriologically confirmed TB. A similar switch to a shorter regimen, if effective and safe, should be assessed for CLHIV.

There are limited data on the effectiveness of IPT on TB incidence in CLHIV. Randomized trials have established the protective effect of IPT for both HIV‐negative children [7] and adults living with HIV [36]. However, a 2018 systematic review of randomized trials of IPT for CLHIV identified only three trials comprising 977 total patients [37], and while point estimates for the effect of IPT all fell on the side of protection, they failed to achieve statistical significance in two of the trials. Studies that evaluated programmatic data have reported protective effects of >80% for 6 months of IPT among CLHIV [31, 32]. In this study, TB incidence among CLHIV who completed a full 6‐month IPT course was four‐fold lower compared to those who never initiated or those who initiated but never completed. These findings thus help to bolster the limited evidence from clinical trials and suggest a protective effect of IPT for CLHIV in programmatic settings.

Our study used routine data collected over several years from eight HIV clinics located within the largest hospitals in the County and was, therefore, suitable for evaluating the outcomes of programmatic IPT in CLHIV in a high burden region in Kenya. Routine data sources are prone to limitations, such as incomplete data, which we minimized by abstracting data from multiple sources, including IPT registers and HIV treatment records. However, we still had several instances of missing data elements on WHO staging (8%), HIV viral load (14%) and IPT initiation dates (3%). Secondly, these routine data sources did not capture some variables that could explain non‐initiation or non‐completion of IPT, such as socio‐economic status, caregiver characteristics (educational status, HIV status, viral load suppression status and TB status), distance from the clinic, INH stockouts and attitudes of providers and caregivers. There are efforts to evaluate these factors in a prospective study. Other than TB status, we did not abstract data on clinical outcomes, such as mortality and loss to follow‐up among IPT non‐initiators, thus these outcomes were not assessed during analysis. TB status was only captured in the medical charts if there was a TB diagnosis but the absence of TB or uncertainty in diagnosis was not outrightly documented in the medical charts.

## 5 CONCLUSIONS

In this study, we observed that while screening for IPT eligibility was high for CLHIV, there were substantial delays and drop‐offs at initiation and completion of IPT. Targeted programmatic interventions are needed to address these drop‐offs from the IPT cascade by ensuring timely IPT initiation after ruling out active TB and enhancing completion of the 6‐month course, especially in high‐volume hospitals. While shorter course TB prevention regimens are becoming more available, they will likely increase completion but potentially not timely initiation. Virally non‐suppressed CLHIV need additional support to initiate and complete IPT and to achieve viral suppression.

## AUTHORS’ CONTRIBUTIONS

DOO, JK, GJ‐S and SML contributed to the conception, design and execution of this study. DOO performed the analyses and drafted the initial manuscript. MABVDS, CMY, EO, JM and DM critically reviewed the paper for analysis and interpretation of the data. All authors revised the manuscript critically and contributed to the final draft and have read and approved the final manuscript.

## COMPETING INTERESTS

The authors have no competing interests to declare.

## FUNDING

Research reported in this publication was supported by the Fogarty International Center of the National Institutes of Health (grant #D43TW009345 awarded to the Northern Pacific Global Health Fellows Program supporting DOO), National Institute of Allergy and Infectious Diseases (NIAID) (NIH/NIAID K23AI120793 to SML) and NIH UL1TR000423 for REDCap.

## DISCLAIMER

The content is solely the responsibility of the authors and does not necessarily represent the official views of the National Institutes of Health.

## Data Availability

Data are available upon reasonable request to the corresponding author.
